# Factors affecting the spiritual rehabilitation of people affected by natural disasters: a systematic review

**Published:** 2024-07

**Authors:** Bayram Nejati-Zarnaqi, Davoud Khorasani-Zavareh, Sanaz Sohrabizadeh, Mohtasham Ghaffari, Siamak Sabour, Fatemeh Nouri, Reza Mohammadi

**Affiliations:** ^*a*^ Department of Health Management and Economics, School of Medicine, Aja University of Medical Sciences, Tehran, Iran.; ^*b*^ Safety Promotion and Injury Prevention Research Center, Shahid Beheshti University of Medical Sciences, Tehran, Iran.; ^*c*^ Air Quality and Climate Change Research Center, Shahid Beheshti University of Medical Sciences, Tehran, Iran.; ^*d*^ Department of Public Health, School of Public Health and Safety, Shahid Beheshti University of Medical Sciences, Tehran, Iran.; ^*e*^ Department of Epidemiology, School of Public Health and Safety, Shahid Beheshti University of Medical Sciences, Tehran, Iran.; ^*f*^ Department of Health in Disasters and Emergencies, School of Public Health and Safety, Shahid Beheshti University of Medical Sciences, Tehran, Iran.; ^*g*^ Division of Family Medicine and Primary Care, Department of Neurobiology, Care Sciences and Society, Karolinska Institutet, Stockholm, Sweden.

**Keywords:** Natural disasters, Spiritual rehabilitation, Spiritual health

## Abstract

**Background:**

Every year, natural disasters in many countries lead to the destruction of infrastructure, loss of assets, and harm to the physical, mental, social, and spiritual health of people. The attention of policymakers and the media is mostly focused on the reconstruction of damaged buildings and the physical rehabilitation and recovery of the injured, while the spiritual rehabilitation of the people affected is often neglected. This study aims to identify the factors influencing the spiritual rehabilitation of people affected by natural disasters.

**Methods:**

This study was conducted using a systematic literature review following the PRISMA guidelines. Relevant studies were extracted from data sources, including MEDLINE (PubMed), Web of Science, Google Scholar, Embase, ProQuest, PsycInfo, Scopus, IranMedex, SID, and ISC. Systematic review studies, key journals, and conference proceedings related to the factors affecting the spiritual rehabilitation of individuals after natural disasters from January 1, 2020 to March 31, 2022 were included. Thematic analysis was used to analyze the obtained data.

**Results:**

Initially, 1,753 studies were identified based on the initial search, and eventually, 22 final studies were included in the study. Based on the thematic analysis results, the factors influencing the spiritual rehabilitation of people affected by natural disasters were classified into four main themes and eleven sub-themes. The main themes included communication with God, strengthening religious beliefs, social participation, and meaning-making. The sub-themes included praying, using supplication, reading the holy book, praising God, believing in the afterlife, understanding the position and characteristics of the world, understanding the divine, participating in religious ceremonies, membership in supportive groups, the meaning of suffering and adversity, and the meaning of death.

**Conclusions:**

The results of this study demonstrate that the connection with the divine (God), strengthening religious beliefs, social participation, and meaning-making are influential factors in the spiritual rehabilitation of affected people after natural disasters. Incorporating these factors in the spiritual counseling and care of the affected people can improve their spiritual health after encountering the destructive effects of natural disasters. These findings can provide valuable insights for managing natural disasters through a holistic approach to the health of affected people, and can guide caregivers in implementing spiritual rehabilitation interventions.

## Introduction

Spirituality has been an integral part of human life from birth to death,^1^ and modern medicine has recently discovered the valuable connection between the body and mind, realizing that illness is not solely limited to physical functioning and the surrounding physical environment.^2^ Numerous studies have confirmed a significant relationship between spirituality and human health.^3,4^ Spiritual health is recognized as a new dimension and the fourth dimension of human health by the World Health Organization.^5^ Despite the ambiguous nature of the concept of spiritual health and the lack of consensus in its definition due to different philosophical perspectives and worldviews,^2,6^ the dimensions of spiritual health can be defined in terms of self-actualization, meaning and purpose in life, inner strength, peace, resilience, self-connection, community and family connection, connection with nature, relationship with the Divine, beliefs, and values.^7^ While it is said in global literature that the concept of higher power can encompass anything sacred, in the perspective of monotheistic religions, the supreme power is solely God with all His inherent attributes such as being all-powerful, compassionate, wise, and just^8^ that in times of worldly challenges and especially during disasters, humans can rely on God and seek His assistance.^9,10^

Facing natural disasters, especially, exposes affected individuals to high levels of stress, leading to severe psychological and spiritual consequences.^11,12^ In such times, humans seek to comprehend the reality of disasters and manage them with the help of the hidden power within spirituality.^1^ Disturbances in spiritual beliefs among disaster survivors, perceiving the disaster as a punishment from God, and feeling rejected by God, diminish their level of spiritual health.^13^ These factors also contribute to psychological disorders and an increased risk of suicide among the affected people.^14,15^ After Hurricane Katrina in 2005, which claimed 1,800 lives and left 370,000 people homeless, many survivors turned to religion and spirituality as a tool to cope with the psychological effects of the hurricane.^16^ Harris demonstrated in his study that disaster survivors seek religious practices such as prayer and worship to achieve peace, which he termed "the search for spiritual support".^17^ After experiencing disasters, individuals may search for spiritual presence through feelings of loss, emptiness, questioning the meaning and purpose of life, experiencing alienation and abandonment, and crying out against injustice.^1^ Following natural disasters, spiritual resources can become an exclusive defense shield^18^ and the only source for managing and accepting the emotions and events that have unfolded.^19^

The rehabilitation of disaster survivors is an essential part of crisis management and aims to reduce the long-term effects of physical, psychological, social, and spiritual damage caused by disasters.^20^ All individuals involved in the spiritual rehabilitation of disaster survivors, including psychologists, psychiatrists, religious organizations, grassroots organizations, and clergy, should be aware of the influential factors in spiritual rehabilitation.^21^ Numerous studies have been conducted on spiritual interventions for providing spiritual care and rehabilitation to hospitalized patients. However, there is a growing recognition of the need for such interventions in the case of natural disaster victims. The environmental conditions that arise after natural disasters, such as the destruction of buildings, loss of property, loved ones, and limited access to medical facilities, create unique needs for those affected by such events. It is crucial to understand the factors that affect the spiritual rehabilitation of disaster survivors after natural disasters to assist disaster management organizations. The findings of this study can contribute to the development and implementation of an operational plan for spiritual rehabilitation in response to natural disasters, completing the recovery phase for disaster survivors. The researcher intends to conduct a systematic review to identify the factors influencing the spiritual rehabilitation of disaster survivors after natural disasters.

## Methods 

The present study was conducted a systematic and thematic content analysis method. In the first phase, a systematic review method was employed to gather relevant articles related to the research objective, following the Preferred Reporting Items for Systematic Reviews and Meta-Analyses (PRISMA) guidelines.^17^ The protocol for this review study is registered in the International Prospective Register of Systematic Reviews (PROSPERO) with the code CRD42021228552. Following the PRISMA protocol, the stages of search strategy, screening, study selection, quality assessment, and data extraction were carried out in order. The stages of study selection, quality assessment, and data extraction were independently performed by two researchers, and in cases of disagreement, decisions were reached through group discussion. Additionally, for the thematic content analysis, the six-step thematic analysis framework by Braun & Clarke^18^ was utilized. The six steps of thematic analysis include familiarization with the data, generating initial codes, searching for themes, reviewing themes, defining themes, and writing a draft.^18^

***Information Sources and Search Strategy***

In the present study, a comprehensive search was conducted using the following databases: MEDLINE (PubMed), Web of Science, Embase, ProQuest, PsycInfo, Scopus, Google Scholar, ISC, SID, Magiran, conference proceedings, key journals, reference lists of selected articles, and systematic reviews. To extract valid keywords, Medical Subject Headings (MeSH) terms, keywords from relevant articles, and consultation with scientific experts were utilized. The valid English keywords used in this study include "Spiritual Health," "Spiritual Rehabilitation", "Spiritual Care", "Spiritual well-being", "Spiritual Beliefs", "Spiritual Support", "Spiritual Therapy", "Spiritual Healing", "Spiritual Concepts", "Spiritual Indicators", "Spiritual Components", "Spiritual Counseling", "Spiritual Intervention", "Faith-Based Health Care", Spiritual, Religion, Faith-Based, Logo, Rehabilitation, Health, Care, well-being, Support, Therapy, Healing, Counseling, Intervention, Disaster, Crisis, Emergency, Catastrophe, Accident, Event. In the first stage, the search syntax was developed based on keywords, operators, and search fields using PubMed, and then the search syntax for other databases mentioned was adapted based on PubMed. The searches in databases were performed from January 1, 2020 to March 31, 2022. The search strategy for the PubMed database is referenced in Table 1. 

**Table 1:** Example of the search for electronic databases according to the Spiritual rehabilitation of affected people after natural disasters.

Database	Syntax
**PubMed**	( ("Spiritual Health"[Title/Abstract]) OR ("Spiritual Rehabilitation"[Title/Abstract]) OR ("Spiritual Care"[Title/Abstract]) OR ("Existential Care"[Title/Abstract]) OR ("Spiritual well-being"[Title/Abstract]) OR ("Spiritual Beliefs"[Title/Abstract]) OR ("Spiritual Support"[Title/Abstract]) OR ("Spiritual Therapy"[Title/Abstract]) OR ("Spiritual Healing"[Title/Abstract]) OR ("Spiritual Concept"[Title/Abstract]) OR ("Spiritual Indicator"[Title/Abstract]) OR ("Spiritual Component"[Title/Abstract]) OR ("Pastoral Care"[Title/Abstract]) OR ("Logo Therapy"[Title/Abstract]) OR ("Faith-Based Care"[Title/Abstract]) OR ("Spiritual Intervention"[Title/Abstract]) OR ("Spiritual Counseling"[Title/Abstract])) AND ( ("Natural Disaster"[Title/Abstract]) OR (Earthquake[Title/Abstract]) OR (Flood[Title/Abstract]) OR (Hurricane[Title/Abstract]) OR (Typhon[Title/Abstract]) OR (Tsunami[Title/Abstract]) OR (Storm[Title/Abstract]) OR (Landslide[Title/Abstract]) OR (Emergency[Title/Abstract]) OR (Crisis[Title/Abstract]) OR (Catastrophe[Title/Abstract])) AND (2000/1/1:2022/12/31[pdat])

***Eligibility criteria***

The inclusion criteria for this study included all studies that focused on spiritual rehabilitation, such as spiritual care, spiritual support, and spiritual counseling, after natural disasters from January 1, 2020 to March 31, 2022. Exclusion criteria encompassed studies related to spiritual rehabilitation after technological disasters (man-made), spiritual rehabilitation of hospitalized patients in hospitals and other healthcare centers, spiritual rehabilitation for individuals with mental disorders, spiritual rehabilitation for mothers of children with congenital abnormalities, spiritual rehabilitation for elderly residents in nursing homes, and other studies that did not specifically address spiritual rehabilitation after natural disaster.

***The selection of studies***

After conducting a search and in order to manage the search results, all articles were imported into the EndNote X7 software. After removing duplicate entries, the titles and abstracts of the remaining articles were screened based on the eligibility criteria, and potentially relevant articles were identified. In the next step, two researchers (BNZ, DKZ) independently read the full texts of the potentially relevant articles and ultimately selected the articles eligible for inclusion in the study.

***Qualitative assessment***

In this stage, two researchers (BNZ, DKZ) independently subjected the selected studies to qualitative assessment. The quality assessment of the chosen studies was conducted using different tools, including the Consolidated criteria for reporting qualitative research (COREQ) for evaluating qualitative studies^22^ the Critical Appraisal Skills Programme (CASP) for evaluating review studies,^23^ and the Modified STROBE (Appendix 1) for evaluating studies that were not amenable to quality assessment using other standard tools.

***Extraction of findings and data analysis***

The required data from the final studies were independently extracted by two researchers (BNZ, DKZ) using a pre-constructed checklist. The checklist included the first author, year of the study, study location, study design, study type, and findings. Thematic content analysis was employed for data analysis. In addition to the headings derived from the studies, the text of the results was also studied and coded. For coding, all codes and initial concepts related to factors affecting the spiritual resilience of disaster survivors, which were extracted as primary data from the studies, were studied line by line and listed. To familiarize themselves with the codes, they were reviewed multiple times, and after that, initial codes were identified. In the next step, the first and second authors (BNZ, DKZ) reviewed all identified codes for similarities and differences, and then similar codes were grouped into a subtheme. In the following stage, subthemes that were conceptually close to each other were grouped to form a theme. Finally, the draft of the designed summaries was discussed among all the authors of this study, and after making modifications, a consensus was reached on the draft findings.

## Results

***Search results***

**Figure 1:**
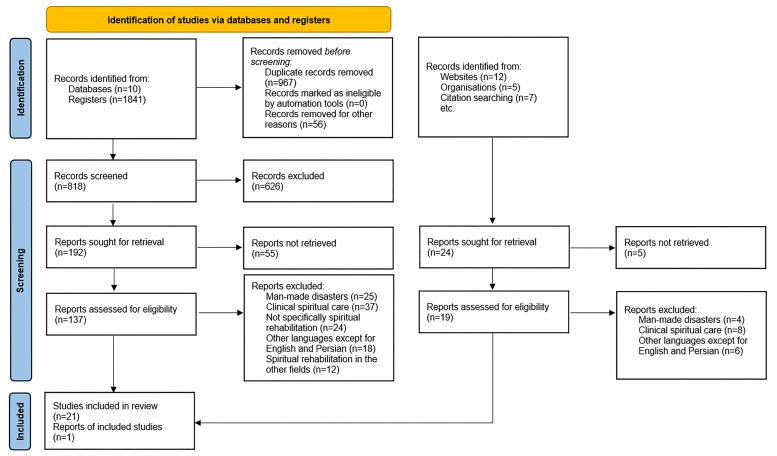
PRISMA flowchart of systematic literature review of factors affecting the spiritual rehabilitation of affected people after natural disasters.

Based on the initial search, 1753 studies were extracted. After removing duplicates, 768 studies were screened based on their titles and abstracts. Following the exclusion of irrelevant studies, the full texts of 117 potentially relevant studies were reviewed. Finally, 22 studies entered the final stage of the study (Figure 1). 

Among the final studies, 12 were conducted in the United States, 1 in China, 1 in Saudi Arabia, 1 in Nigeria, 1 in the Netherlands, 1 in Malaysia, 2 in Iran, 1 in Denmark, 1 in Canada, and 1 in France. Most of the studies had a qualitative and descriptive design. Based on the qualitative assessment of the studies, 12 studies were deemed to have good quality, while 9 studies were of average quality. The details of the selected articles are provided in Table 2. 

**Table 2:** Specifications of the extracted articles in the systematic review on identifying factors affecting the spiritual rehabilitation of affected people after natural disasters.

Number	First Author / Year of Publication	Place	Publication Type	Study Design	Finding of Article
1	Patric R. Spence (2007) ^24^	USA	Journal article	Survey	Faith is one of the factors that contribute to spiritual support, and prayer can be one of the coping strategies.
2	Joseph U. Almazan (2018) ^25^	Saudi Arabia	Journal article	Qualitative	Praying and engaging in religious practices can enhance the resilience of survivors.
3	John P. Reed (1977) ^26^	USA	Journal article	Descriptive	Hope; Holding discussions; participating in religious ceremonies; Empathy; Theology
4	Leila Mohammadinia (2018)^27^	Iran	Journal article	Review	Belief in God; Prayer and worship; Reading holy books; Engaging in religious practices; Participating in religious ceremonies; Self-confidence Derived from trust in God; Honesty
5	Loren D. Marks (2015) ^28^	USA	Book section	Qualitative	Prayer serves as a means of coping, Seeking assistance, and guidance from God.
6	AbdulGafar Olawale Fahm (2019) ^9^	Nigeria	Journal article	Descriptive	Reliance; Patience; Gratitude
7	Allison Kestenbaum (2018) ^29^	USA	Journal article	Qualitative	Understanding of God; Meaning-making; Hope; Understanding the concept of life and the afterlife; Gratitude; Life purpose
8	Amy L. Ai (2013) ^30^	USA	Journal article	Mixed Method	Hope; Faith in God; Religious beliefs; Prayer
9	Christina Ta (2011)^31^	USA	Journal article	Qualitative	Support from religious communities; Religious practices such as prayer; Participation in religious ceremonies; Spiritual beliefs
10	David N. Entwistle (2018) ^32^	USA	Journal article	Descriptive	Knowing God; Understanding the role of disaster in the world; Prayer; Empathy; Taking advantage of other family members and friends
11	Kelly M. Trevino (2007) ^33^	USA	Journal article	Letter	Communication with God; Meaning of life; Support from religious leaders and groups; God’s position
12	Lei Sun (2017) ^34^	China	Journal article	Review	Understanding the causes of disasters; Knowing God; Making life purposeful; Hope; Communication with the superior power
13	Melissa A. Heath (2020) ^35^	USA	Journal article	Descriptive	Prayer; Knowing God's position; The purpose of life; Knowing death; Participating in religious ceremonies; Life after death
14	Rafeah Saidon (2020)^11^	Malaysia	Journal article	Review & Qualitative	Remembrance of God; Prayer; Congregational prayer; Recitation of the Quran; Religious acts
15	Nejati-Zarnaqi Bayram (2022) ^36^	Iran	Journal article	Qualitative	Correcting victims’ perspectives; Describing God’s characteristics; Seeking help from God; Strengthening spiritual beliefs; Psychological factors; Tranquility factors
16	Stephen Muse (2007)^37^	USA	Journal article	Qualitative	Communication with God; Prayer; The meaning of death
17	Rasmus Dahlberg (2015) ^38^	Denmark	Journal article	Descriptive	The meaning of suffering; The purpose of life; Prayer; Knowing God; Forgiveness; Prayer; Participation in religious ceremonies
18	Rosemary Chinnici (1985) ^39^	USA	Journal article	Qualitative	Prayer; Communication with God; Hope; Participation in funeral ceremonies
19	Saleem Khaldoon Al-Nuaimi (2020)^40^	Canada	Journal article	Descriptive	Patience; Repentance; Prayer; Remembrance of God; Prayer - Expression of destiny; The meaning of suffering; Understanding the reality of death
20	Tammy L. Henderson (2001) ^41^	USA	Journal article	Mixed Method	Hope for the future; Music; Reading the Bible; Communication with friends; Prayer; Communication with God; Participation in spiritual ceremonies; Reliance on God
21	Martin W. Feldbush (2007)^42^	USA	Journal article	Letter	Giving hope; The meaning of life; Knowing God; Social support; Knowing the afterlife; Prayer
22	Judite Blanc (2015) ^43^	France	Journal article	Cross-Sectional	Knowing God; The causes of disasters; The meaning of suffering in life; The purpose of life

***Thematic Content Analysis***

Based on a systematic and thematic content analysis review, the factors affecting the spiritual rehabilitation of natural disaster survivors were classified into four main themes and eleven sub-themes. The themes include communication with God, strengthening religious beliefs, social participation, and finding meaning. The sub-themes include praying, using supplication, reading the holy book, praising God, believing in the afterlife, understanding the position and characteristics of the world, understanding the divine, participating in religious ceremonies, membership in supportive groups, the meaning of suffering and adversity, and the meaning of death (Table 3).

**Table 3:** Factors affecting the spiritual rehabilitation of affected people after natural disasters based on a systematic review and thematic content analysis.

Theme	Sub-themes	Sample Code
**Communication with God**	Praying	• Tranquility through prayer
		• Prayer is a means of communication with a supreme power
	Using supplication	• Supplication means communication with God
		• Asking for help with supplication
	Reading the scriptures	• Reading Quran
		• Reading the Bible
	Praising God	• Get peace of mind through praising God
		• Remembering God through praising God
**Strengthening religious beliefs**	Believing in the afterlife	• Understanding the concept of life and the hereafter
		• Belief in life after death
	Understanding the position and characteristics of the world	• The purpose of life
		• The duty of man in the world
	Understanding the divine	• Trust in God
		• Faith in God
**Social participation**	Participating in religious ceremonies	• Participating in congregational prayers
		• Participation in funerals and burials
	Membership in supportive groups	• Help in settling the affected people
		• Helping to remove the debris
**Meaning-making**	The meaning of suffering and adversity	• Knowing the causes of disasters
		• Understanding the reason for human suffering
	The meaning of death	• Knowing death
		• Man is not destroyed by death

## Discussion

Based on the findings of the current review study, various factors such as communication with God, strengthening religious beliefs, social participation, and meaning-making can play a significant role in the spiritual recovery of disaster survivors. The present systematic review showed that communication with God through prayer, supplications, reading holy books, and reciting praises can have an impact on the spiritual recovery of disaster survivors. Jerry stated in his study that prayer and supplications play an important role in supporting and protecting individuals affected by disasters and should be considered.^44^ Prayer forms a connection between the survivor and God as a supreme power. In fact, the survivor, through prayer, seek to invite God into their life, and through this relationship, they can share their needs, emotions, and fears with God.^45^ Praying enhances the ability of survivors to cope with the effects of disasters and creates a sense of assurance and hope that every event has been caused by God.^46^ Another method of spiritual connection between the survivor and God is reading holy books such as the Quran^47^ and the Bible,^48^ which can serve as a solution to enhance the level of spiritual health of disaster survivors. Therefore, it is recommended that providing the necessary facilities for prayer and religious practices according to the beliefs of the affected region be given priority.

According to the present study, it is essential for disaster survivors, especially those who have lost loved ones, to understand the true meaning of death for spiritual recovery. Guler's study showed that individuals who comprehend the reality of death and consider it as a part of their life experience a greater sense of meaning in their lives and have less grief when facing the death of their loved ones, going through the natural cycle of mourning.^49^ Many divine religions view death not as annihilation, but as the beginning of an evolutionary journey into a broader realm.^50^ Survivors deeply affected by disasters need to realize that the death of their loved ones was not selective act, and all humans will eventually join them through various paths after a few moments. ^51^ Realizing the reality of death in human lives allows the deeply affected survivors to maintain their spiritual health while reducing the anxiety surrounding death.^52^ It is recommended to address disaster survivors’ perceptions of the meaning of death and the afterlife in their spiritual recovery from natural disasters.

The disruption of spiritual health in disaster survivors, especially in the case of natural disasters,^53^ can stem from attributing blame to God for the occurrence of disasters. This may be due to a belief in God's inability to control them or as a punishment from God through disasters.^54^ After natural disasters, many survivors overlook the laws governing this world and the cause-and-effect relationships within it, directing all their emotional burdens onto God.^55^ Survivors of natural disasters often turn to God either complain or seek refuge in Him. Therefore, it is essential to explain to survivors that natural disasters are random occurrences, similar to all the good and bad events that happen in this world based on cause-and-effect relationships. In this regard, describing the attributes of God, such as His compassion, power, wisdom, and benevolence, can have positive effects.

In the face of hardships, including natural disasters, people are compelled to seek refuge in their spiritual beliefs to find support, guidance, and ways to cope with these difficulties.^56^ If humans can find meaning in every affliction and suffering that befalls them in this world, they can endure it more easily and adapt to it.^57^ Just as professional athletes find meaning in the hardships they endure, being titled as champions, this meaning not only makes their training difficulties bearable but also makes them sweet and valuable. Assigning meaning to the hardships encountered by humans has been one of the primary functions of religion,^58^ and religion can help its followers comprehend the meaning of the calamities that occur in this world, as well as the worldly consequences and rewards of enduring hardships resulting from such calamities.^59^

Based on the findings of the present study, social participation of disaster survivors, such as participating in religious ceremonies and joining support groups, can have an impact on their spiritual recovery. A study on earthquake survivors of the 2009 Indonesian earthquake revealed that participating in congregational prayers and religious ceremonies helped reduce their grief, sorrow, and depression while also strengthening their sense of belonging in the affected community.^46^ Connection with others^60^ and solidarity with the community^61^ are dimensions of spiritual health, and the Huber study demonstrated that the type of friendship and solidarity are directly correlates with spirituality.^62^ Connecting with others can help maintain and enhance individuals' spiritual health and contribute to a better understanding of meaning and life purpose.^63^ It is recommended to form teams of survivors and involve them in activities such as debris removal, housing, distribution of aid, and funeral ceremonies to facilitate their participation.

## Conclusion

The results of the present study demonstrate that various factors, including communication with God, strengthening religious beliefs, social participation, and meaning-making, are effective in the spiritual recovery of disaster survivors. Knowing and connecting with God, as the supreme power and source of support in post-disaster situations, can serve as a refuge and support for the affected individuals. Meaning-making for the loss of loved ones and the suffering endured by survivors of natural disasters can ease their hardships and instill hope for the future. The findings from this study can be utilized for policy-making and planning toward the spiritual recovery of disaster survivors by considering the culture and religious/spiritual beliefs prevailing in the affected community. The effective factors extracted from this study can be used in the Mitigation phase for educating and enhancing the level of the spiritual health of individuals in the community before the occurrence of disasters and in the Preparedness phase for developing guidelines and protocols related to spiritual recovery, as well as training intervention providers in spiritual recovery.

**Acknowledgment:**

This project is supported both financially and officially by Shahid Beheshti University of Medical Science, Tehran, Iran.

**Author Contributions:**

BNZ, DKZ, and S Sabour designed the study. BNZ, S Sohrabizadeh and DKZ collected the data. MGH, RM, FN and BNZ were involved in data analysis. BNZ, DKZ, S Sohrabizadeh, S Sabour and RM drafted the manuscript. All authors reviewed and approved the final manuscript.
